# Rational approaches for engineering novel functionalities in carbon-carbon bond forming enzymes

**DOI:** 10.5936/csbj.201209003

**Published:** 2012-10-02

**Authors:** Perrin Baker, Stephen Y. K. Seah

**Affiliations:** a Department of Molecular and Cellular Biology, University of Guelph, Guelph, Ontario Canada, N1G 2W1

## Abstract

Enzymes that catalyze carbon-carbon bond formation can be exploited as biocatalyst for synthetic organic chemistry. However, natural enzymes frequently do not possess the required properties or specificities to catalyze industrially useful transformations. This mini-review describes recent work using knowledge-guided site-specific mutagenesis of key active site residues to alter substrate specificity, stereospecificity and reaction specificity of these enzymes. In addition, examples of *de novo* designed enzymes that catalyze C-C bond reactions not found in nature will be discussed.

## Introduction

Enzymes from the lyase (EC 4.1), ligase (EC 6.4) and hydrolase (EC 3.1, 3.7) classes have been explored for organic synthesis of value-added compounds and pharmaceuticals due to their ability to catalyze carbon-carbon bond formation (for reviews please see [1–4]). Natural enzymes however, do not exist for many industrially important transformations and therefore there is practical interest in tailoring the enzymes for novel functionalities. This can be accomplished through directed evolution approaches, involving random mutagenesis followed by screening of resultant enzyme variants for desired properties [5–8]. However, this approach tends to be labour intensive and the requirements for a screening method can limit the type of reactions that can be efficiently selected. Rational design approaches, involving site-specific mutagenesis of key residues of the enzyme is an alternative means to alter enzymes’ properties or function. This often requires an intimate knowledge of the enzyme's structure and its relationship to function. In some cases, when functional significance of an amino acid is unknown, “semi-rational” approaches can be employed, targeting a specific amino acid for multiple mutations. There have been a number of recent examples of successful rational or semi-rational design of C-C bond forming enzymes, which will be the focus of this review. Some exciting developments on the *de novo* rational design of enzymes to catalyze novel C-C bond forming chemistries are also presented.

## Alterations to Substrate Specificity

One of the main limitations of enzymes as catalysts for synthetic chemistry is that natural enzymes can only transform a limited range of substrates. In the cases discussed below, modifications of enzymes’ substrate binding sites could have profound effects on substrate specificity.

While many enzymes have evolved to accept phosphorylated substrates, synthetic chemists rarely want to prepare phosphorylated products. 2-deoxyribose-5-phosphate aldolase (DERA) has been modified to increase its preference for non-phosphorylated substrates. DERA is unique among aldolases as it catalyzes the reversible asymmetric aldol addition reaction of two aldehydes, acetaldehyde and D-glyceraldehyde-3-phosphate, to generate D-2-deoxyribose-5-phosphate [9]. Using the 1.05 Å crystal structure of DERA in complex with its natural substrate, two positively charged residues (K172 and R207) and three neutral active site residues (G205, S238, S239), located in the phosphate binding pocket were replaced with negatively charged aspartate or glutamate to change the enzyme specificity from the utilization of a negatively charged phosphorylated substrate to a non-phosphorylated neutral D-2-deoxyribose (DR) substrate. In each of the mutants, specificity constants (*k*_*cat*_ */K*_*m*_) for the natural substrate were reduced by 11-fold to 10^8^-fold. Specificity constants for DR were only improved in the S238D variant by 2.5-fold, possibly due to favorable hydrogen bonding interactions between the aspartate side chain carboxylate at position 238 and the terminal hydroxyl of D-glyceraldehyde. Interestingly, the S238D variant was able to catalyze a novel sequential aldol reaction using 3-azidopropinaldehyde, which is not a substrate for the wild-type enzyme, as the first acceptor and two molecules of acetaldehyde as donor to form an azidoethyl pyranos. This is a key intermediate in the synthesis of Lipitor™, a statin drug used for lowering blood cholesterol.

**Figure 1:**
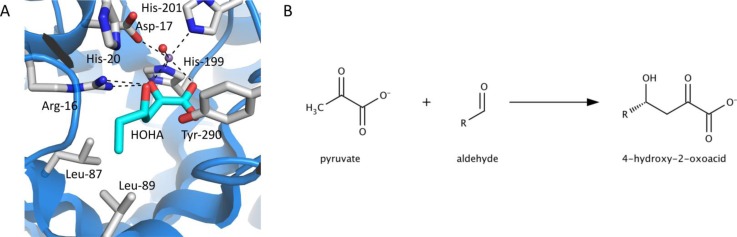
**Active site of pyruvate aldolase and the reaction it catalyzes**. (A) Active site of DmpG, an ortholog of BphI, containing the modeled substrate 4-hydroxy-2-oxohexanoate (HOHA). The alkyl chain of the 4-hydroxy-2-oxoacid extends towards Leu-89. Carbon atoms of the substrate are colored cyan, and the residues in the substrate binding sites are colored gray. Numbers correspond to residues in BphI. (B) The enzyme, BphI, catalyzes the aldol addition reaction between pyruvate and an aldehyde. R= CH3 and C5H11 for acetaldehyde and pentaldehyde, respectively.

Specificity of an enzyme can also be altered by steric modifications to the active site of an enzyme. The substrate specificity of BphI, a class II pyruvate aldolase [10], was altered using the crystal structure of an orthologous aldolase, DmpG (PDB 1NVM), as a guide [11]. The natural substrate 4-hydroxy-2-oxopentanoate and 4-hydroxy-2-oxoacids containing unfunctionalized aldehydes of different lengths were modeled into the active site of DmpG [12] (Figure 1). Leu-87, which was proposed to be located proximal to the C4-methyl of 4-hydroxy-2-oxopentanoate and Leu-89, located at the opposite side of the active site from the pyruvvl moiety in BphI were replaced with smaller alanine residues. The replacement of Leu-87 with alanine reduced the specificity constants of the enzyme toward acetaldehyde by 32-fold while maintaining the same specificity constant for propionaldehyde as in the wild-type enzyme. The L89A variant exhibited similar *k*_cat_/ *K*_m_ values for acetaldehyde and propionaldehyde as in the wild-type enzyme, however specificities toward butyraldehyde, pentaldehyde, and hexaldehyde increased dramatically. For example, the specificity of the L89A variant for pentaldehyde was ∼50-fold higher than that of the wild type, making it a more efficient enzyme (k_cat_/ *K*_m_ = 23.3 ± 2.8 M^-1^ s^-1^) for utilization of this longer chain aldehyde than the wild-type enzyme for its natural substrate, acetaldehyde (k_cat_/ *K*_m_ = 13.4 ± 0.8 M^-1^ s^-1^). These results demonstrate that the alkyl moiety of the aldehyde substrate extends towards Leu-89, and reducing the side chain size of this residue can create an enzyme with increased specificity for longer chain aldehydes.

Dihydroxyacetone (DHA) and dihydroxyacetone phosphate (DHAP)-dependent aldolases have been extensively utilized in organic synthesis because they catalyze stereoselective aldol additions leading to the formation of useful chiral polyhydroxylated structures [13]. A number of studies have successfully utilized rational and semi-rational approaches, guided by X-ray crystal structures, to alter the substrate specificity through mutations in the phosphate-binding and other active site residues [14, 15].

Of special attention is FucA, a L-fuculose-1-phosphate aldolase from *E. coli*. DHAP aldolases, such as FucA, are specific for the DHAP donor substrate but can accept structurally diverse aldehyde acceptors. In spite of this diverse specificity towards the aldehyde acceptors, the enzyme does not effectively catalyze reactions involving *N*-Cbz-amino aldehydes (Cbz = benzyloxycarbonyl) containing α-branched substituents to produce polyhdroxylated pyrrolizidines glycosidase inhibitors. Using the crystal structure of FucA [16], Clapés and colleagues targeted Phe-131 and Phe-206 that compose a hydrophobic wall at the binding site for the natural acceptor substrate, L-lactaldehyde. Single alanine mutations of these residues were created to effectively remove these aromatic residues that sterically hinder the productive binding of branched α-substituted *N*-Cbz-amino aldehydes. While F131A and F206A variants exhibited a 5 to 100-fold reduction in specific activity for the natural reaction, the F131A was observed to catalyze the aldol addition reactions of DHAP to various *N*-Cbz-amino aldehyde derivatives 4 to 25-fold higher than that of wild-type FucA [17]. Moreover, F131A displayed aldol activity with (*S*)- and (*R*)-*N*-Cbz-prolinal, whereas no detectable activity was observed in wild-type FucA for the (*R*)-enantiomer. Computational binding models and molecular dynamic simulations further indicated that the phenyl group of Cbz can occupy the space generated by the F131A and F206A and that in F131A, the Cbz-phenyl group is stabilized by a π-cation interaction with the charged ɛ-amine of Lys-205. This interaction would therefore increase binding affinity of these substrates relative to the wild-type enzyme. Thus, a single point mutation effectively eliminated a steric constraint, allowing for a non-natural π-cation interaction, allowing for the synthesis of novel polyhdroxylated pyrrolizidines of the hyacinthacine and alexine types.

Another such aldolase which has received considerable attention is the class I D-fructose-6-phosphate aldolase, FsaA from *E. coli*. Its catalytic potential arises from the fact that the enzyme can catalyze the asymmetric synthesis of polyhydroxylated compounds utilizing unphosphorylated dihydroxyacetone (DHA) instead of the more expensive DHAP. The aldolase catalyzes the reversible aldol addition of DHA, hydroxyacetone (HA) and hydroxybutanone (HB) with a variety of acceptor aldehydes. To improve the enzyme's specificity for DHA by allowing hydrogen bonding to occur with the C-1 hydroxyl of this substrate, an A129S variant was constructed. The *k*_cat_/ *K*_m_ values for this variant towards DHA increased by 17-fold [18]. It is thought that the serine stabilizes the DHA donor substrate and may also be involved in the stabilization of the Schiff-base intermediate [19]. Despite the ability for the enzyme to utilize a number of donor substrates, the enzyme has limitations in its acceptor substrate tolerance. In an attempt to rationally design the enzyme to complete the aldol addition of DHA or HA to α-substituted aminoaldehydes, such as (*S*)- and (*R*)- *N*-Cbz-alaninals, Clapés and co-workers identified a putative residue in the active site thought to be involved in acceptor substrate-binding. Substitution of Ala-165 with glycine was envisaged to create more space in the active site to accommodate the C-α substituents of the aldehyde acceptors. Indeed, the variant catalyzed aldol addition reaction using HA and DHA as the donor and α-substituted aminoaldehydes as the acceptor with specific activities 4 to 175-fold higher than the wild-type enzyme [20]. Combining the two mutations, A129S and A165G, improved the activity of the enzyme in catalyzing the aldol addition of DHA and HA to selected aldehydes. These mutations did not result in changes in the stereochemical outcome of the reaction suggesting that the active site is not substantially altered.

**Figure 2:**
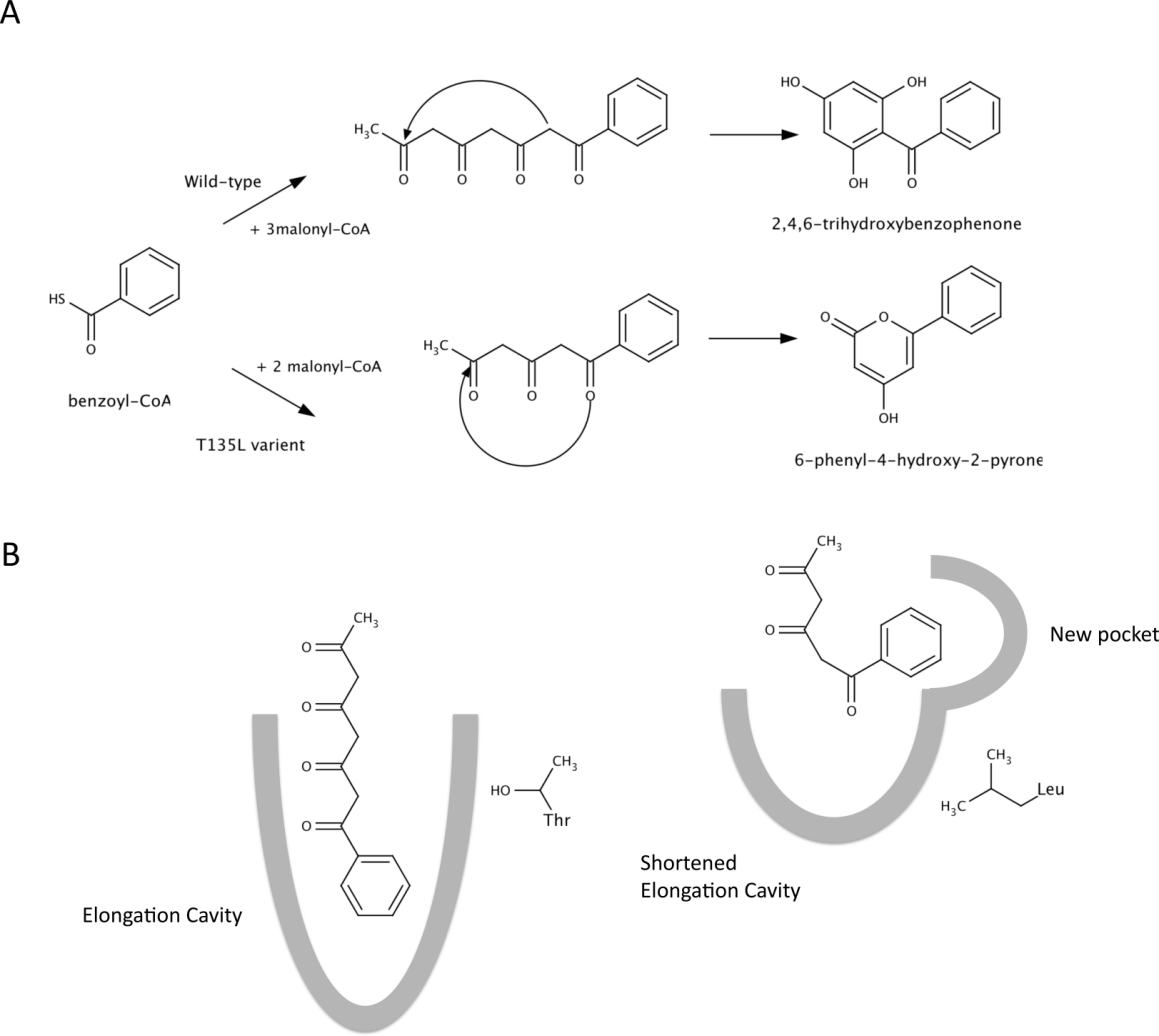
Postulated mechanism of wild-type BPS and the T135L variant. (A) The wild-type enzyme catalyzes the condensation of benzoyl-CoA with three malonyl-CoAs to form a tetraketide which cyclizes to 2,4,6-trihydroxybenzophenone. The T135L variant is only able to catalyze the condensation of benzoyl-CoA with two malonyl-CoAs, forming a triketide which cyclizes to 6-phenyl-4-hydroxy-2-pyrone. (B) Differences in active site structure result in altered product specificity. In the wild-type enzyme the benzene ring of benzoyl-CoA projects into the elongation tunnel as acetyl units from malonyl-CoA are added. This tunnel is open due to hydrogen bonding between Thr-135 and the backbone amides of Gln-165 and Gly-166. In the T135L variant, leucine projects into the tunnel, resulting in a steric block while eliminating the hydrogen-bonding with Gln-165 and Gly-166 and resulting in the formation of a new pocket. Favorable hydrophobic interactions between Leu-135 and the benzene ring of benzoyl-CoA result in a new orientation of substrate binding and elongation in the T135L variant.

Benzophenone derivatives constitute a class of secondary plant metabolites that include a number of pharmacologically active constituents, including garcinol and gambogic acid. The formation of the carbon skeleton of these benzophenone derivatives is catalyzed by benzophenone synthase (BPS), a type II polyketide synthase. In *Hypericum androsaemum*, BPS catalyzes the iterative condensation of benzoyl-CoA with three molecules of malonyl-CoA to give a linear tetraketide intermediate, which subsequently cyclizes into 2,4,6-trihydroxybenzophenone by Claisen condensation [21]. The enzyme also synthesizes small amounts of 6-phenyl-4-hydroxy-2-pyrone (phenylpyrone). Using molecular modeling based on the crystal structure of chalcone synthase (59.1% sequence identity with benzophenone synthase) (PDB 1CGZ) [22] as a guide, an attempt was made to alter substrate specificity. In the wild-type enzyme, Thr-135 forms hydrogen bonds with the backbone of Gly-166 (Figure 2). A T135L variant was created such that the leucine side chain protrudes into the elongation cavity and at the same time disrupting the hydrogen bond formed with Gly-166. This resulted in the opening of a new pocket within the active site to accommodate a phenyl group of the substrate. Relative to the wild-type enzyme, the variant catalyzed the addition of only two acetyl groups from malonyl-CoA to the benzoyl starter unit forming phenylpyrone. The catalytic efficiency (*k*_cat_/ *K*_m_) for the variant was almost unchanged relative to the wild-type enzyme as a 8-fold reduction in *k*_cat_ was offset by a 8-fold reduction in *K*_m_. When 3-hydroxybenzoyl-CoA is used as the starter substrate, the wild-type enzyme catalyzes the stepwise addition of three acetyl groups to give a tetraketide which cyclizes into 2,3’,4,6-tetrahydroxybenzophenone. The T135L variant however is almost inactive with 3-hydroxy-benzoyl CoA as a starter substrate as the variant eliminates H-bonding between the enzyme and the 3-hydroxyl group of the aryl moeity. The 3-hydroxybenzoyl-primed triketide therefore becomes trapped in the new pocket of the T135L variant. Thus, the T135L substitution changes both the substrate and product specificity of BPS.

## Modifying enantiomeric selectivity

One of the main advantages of using enzymes as catalysts for C-C bond formation is their stereoselectivity, which is important for synthesis of pharmaceutical drugs. Examples of rational design of stereochemical control in C-C bond forming enzymes are discussed below.

**Figure 3:**
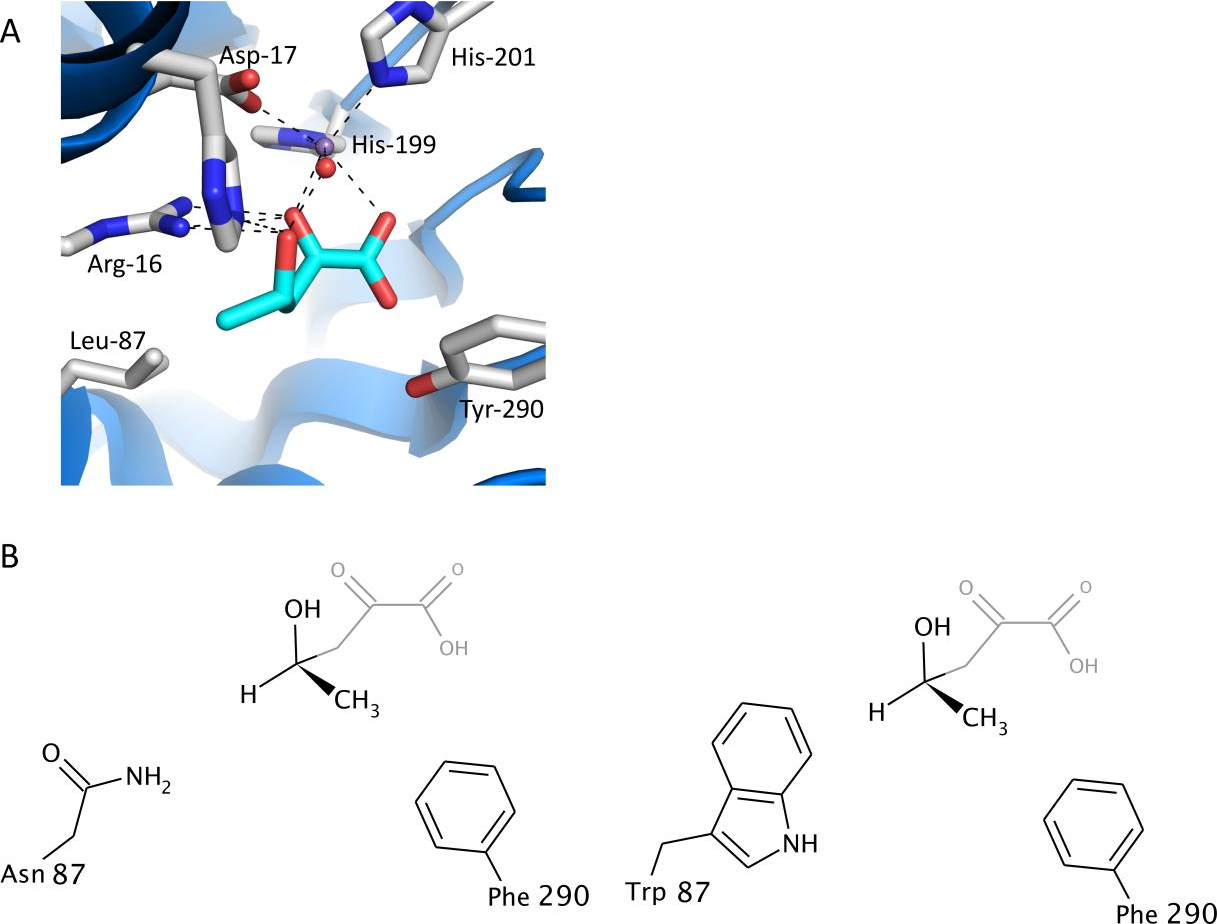
Engineering stereospecificity in BphI. (A) Model of 4(*S*)-hydroxy-2-oxopentanoate in the active site of DmpG, an ortholog of BphI. The chiral C4 carbon is positioned between Leu-87 and the phenolic oxygen of Tyr-290. Carbon atoms of the substrate are colored cyan, and the carbon atoms of residues in the substrate binding sites are colored gray. Numbers correspond to residues in BphI. (B) To allow for binding of the 4-methyl in the 4(R)-enantiomer, the phenolic oxygen was removed with a Y290F variant. At the same time, Leu87 was replaced with asparagine and tryptophan so that binding of the 4(S) enantiomer is disfavoured.

The class II pyruvate aldolase BphI exhibits strict stereochemical control as it can only utilize or produce the (4*S*) enantiomer of 4-hydroxy-2-oxopentanoate [23]. Y290F and Y290S variants were found to lose stereochemical control and were able to catalyze aldol cleavage reactions of both (4*S*) and (4*R*) enantiomers of 4-hydroxy-2-oxopentanoate with similar catalytic efficiencies [12]. This is consistent with the proposal that the *p*-hydroxyl of Tyr-290 in the wild-type enzyme sterically restricts the binding of the (4*R*)-enantiomer and its removal in the Y290F or Y290S variants enables the enzyme to utilize this enantiomer. Modeling of the 4-hydroxy-2-oxopentanoate in the active site of an orthologous enzyme placed the C4 chiral carbon between Leu-87 and Tyr-290 (Figure 3A) [12]. Replacement of Leu-87 with the polar asparagine (L87N) and the bulky tryptophan (L87W) residue were then attempted to create unfavorable polar-hydrophobic interactions or to introduce steric constraints to prevent binding of (4*S*) enantiomer. The double mutants, L87N/Y290F and L87W/Y290F were observed to catalyze the cleavage of (4*R*)-hydroxyl-2-oxopentanoate and not the (4*S*) enantiomer [24] (Figure 3B). In these double variants, pyruvate binding was unaffected and the enzyme maintained stereospecificity for substrates of various lengths that is opposite to that of the wild-type enzyme.

**Figure 4:**
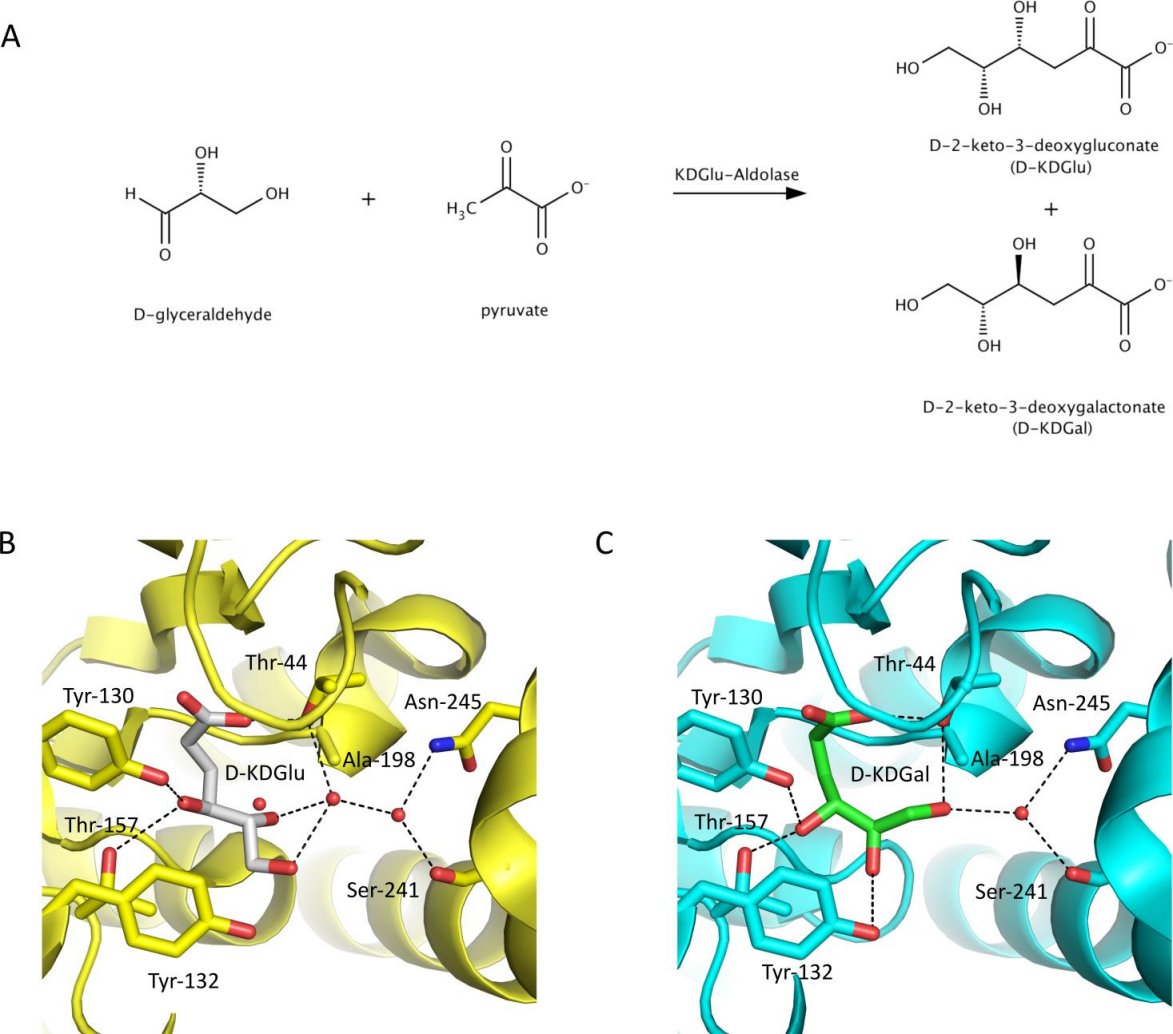
**Engineering stereochemical control in 2-keto-3-deoxygluconate aldolase**. (A) The wild-type aldolase catalyzes the nonstereoselective aldol addition reaction between pyruvate and D-glyceraldehyde. (B) X-ray structure of 2-keto-3-deoxygluconate aldolase from *Sulfolobus sofataricus* bound with D-KDGlu (PDB 1W3N). D-KDGlu makes water mediated H-bonding interactions with the enzyme and the C6-OH is stabilized by direct H-bonding with Tyr-132 and water mediated hydrogen bonding with Thr-44, Ser-241 and Asn-245 (C) X-ray structure of 2-keto-3-deoxygluconate aldolase from *Sulfolobus sofataricus* bound with D-KDGal (PDB 1W3T). The C5-OH of bound D-KDGal hydrogen bonds directly with Tyr-132 and the C6-OH interacts directly with Thr-44. Structures are depicted in cartoon with active site residues shown as sticks.

Stereospecificity of a class I aldolase, 2-keto-3-deoxygluconate aldolase, has also been improved by rational design [25]. This enzyme exhibits broad specificity for the aldol addition reaction between pyruvate and non-phosphorylated aldehydes [26]. The wild-type enzyme exhibits poor diasterocontrol in many of its aldol reactions, including the reaction of its natural substrates, pyruvate and D-glyeraldehyde producing a 55:45 racemate of D-2-keto-3-deoxygluconate (D-KDGlu) and D-keto-3deoxy-galactonate (D-KDGal) (Figure 4A). X-ray crystal structures of imine covalent complexes of this aldolase with pyruvate, D-KDGlu and D-KDGal have revealed that the carboxylate and C4-OH of D-KDGlu and D-KDGal are stabilized by common interactions. However, the C5-OH of D-KDGlu makes water mediated H-bonding interactions with the enzyme and the C6-OH is stabilized by direct H-bonding with Tyr-132 and water mediated hydrogen bonding with Thr-44, Ser-241 and Asn-245 (Figure 4B). In comparison, the C5-OH of D-KDGal H-bonds directly with Tyr-132 and the C6-OH interacts directly with Thr-44 (Figure 4C). This indicates that binding of D-KDGlu is highly dependent on a conserved H-bond network composed of water molecules. Although Thr-157 plays a role in H-bonding to the C4-OH of both diastereoisomers, it is more important for binding of D-KDGal (distance between C_β_-hydroxyl of the threonine side chain and C4-OH of D-KDGal and D-KDGlu is 2.47 Å and 3.07 Å, respectively). Thus, replacement of Thr-157 with residues of larger side chains, T157C and T157F, led to improved stereocontrol for D-KDGlu with 75% dr and 79% dr, respectively. To improve the stereochemical control for D-KDGlu, Tyr-132 that interacts directly with the C5-OH was replaced with valine. The Y132V variant catalyzed the selective formation of D-KDGlu in 79% dr. The Y132V/T157C and Y132V/T157F double variants were able to catalyze the formation of D-KDGlu with 91% dr and 93%, respectively. A separate set of variants was created to increase the selectivity of the enzyme for KDGal by reducing the H-bonding network required for stabilization of KDGlu. An A198L/T157G variant resulted in a small improvement in diastereocontrol, resulting in a preference for D-KDGal with a 72% dr. Asp-181, which is oriented towards Leu-198 was subsequently substituted with Gln to lengthen this polar amino acid and further direct the hydrophobic side chain of Leu-198 towards C5-OH of D-KDGlu, thus destabilizing the interaction with this substrate. In addition, the Leu-198 side chain is thought to stabilize the C1-C3 hydrophobic face of the D-glyceraldehyde conformer of D-KDGal. Indeed, the resulting triple variant A198L/T157G/D181Q was able to catalyze the diastereoselective formation of D-KDGal with 88% dr. However, improved stereoselectivity came with a >70-fold decrease in *k*_cat_.

**Figure 5:**
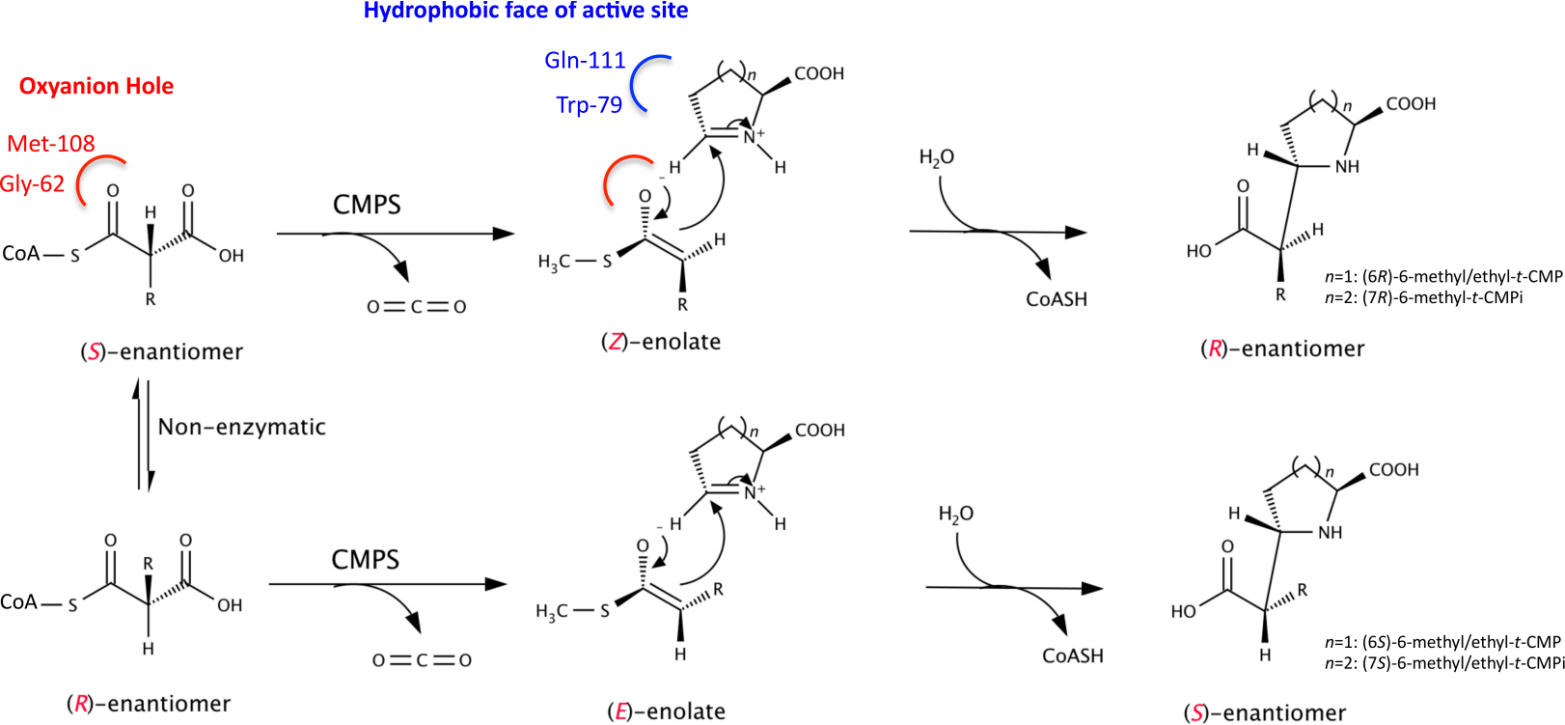
Proposed mechanism for the CMPS-catalyzed reaction. The decarboxylation of either alkylmalonyl-CoA enantiomer results in the formation of a either (*E*)- or (*Z*)-enolate intermediate, which reacts with an imine to give a specific diastereomer of carboxymethyl-substituted *N*-heterocycles.

Carboxmethylproline synthases (CMPSs) belong to the crotonase superfamily of enzymes that catalyze an early step in carbapenem antibiotic biosynthesis [27]. Under standard assay conditions the CMPS from *Pectobacterium carotovorum*, known as CarB, catalyzes the reaction between L-GHP (an equilibrium mixture of L-glutamate semialdehyde, 5-hydroxyl-L-proline and L-pyrroline-5-carboxylate) with a C2 epimeric mixture of methylmalonyl-CoA to give a ∼55:45 mixture of (6*R*)- and (6*S*)-epimers of 6-methyl carboxymethylproline (6-methyl-*t*-CMP) [28]. While no crystal structure of CarB in complex with L-GHP is present, previous knowledge that the enolate anion formed during the reaction is likely bound in an oxyanion hole composed of the backbone amides of Met-108 and Gly-62 led to targeting of these residues for site-specific mutagenesis in an attempt to introduce stereochemical control (Figure 5). Residues, Gln-111 and Trp-79 in CarB, thought to be involved in L-GHP binding, were also replaced by mutagenesis. Substitution of Met-108 in CarB with amino acid residues containing a β-branch, such as valine or isoleucine, resulted in an enzyme that preferentially produced the (6*R*) products (≥95:5 for 6*R*:6*S*) possibly due to steric clash between the β–methyl and reaction intermediates that result in the formation of the (6*S*) alkyl substituent product. Substitution of Trp-79 with alanine or phenylalanine on the other hand resulted in variants that preferentially produce the (6*S*) enantiomer product with ≥5:95 for 6*R*:6*S* [29]. It was observed that W79F and W79A variants also have a ∼10-fold increase in catalytic rate relative to the wild-type enzyme. While the chemical basis behind the diastereoselectivity exhibited in the variants mentioned is not clear, it is anticipated that these substitutions may influence the binding of alkylmalonyl-CoA and the catalytic rates of the two half reactions.

## Engineering novel functionalities

Comparison of enzymes that share similarities in structure or reaction chemistry can be useful to engineer enzymes with novel functions.

Alanine racemase and L-threonine aldolase are pyridoxal 5’-phosphate (PLP)-dependent enzymes that are evolutionarily unrelated and have completely different tertiary and quaternary structures, substrate preferences and reaction specificities [30]. However both enzymes form aldimine intermediates between PLP and their respective substrates, alanine or β-hydroxy-α-amino acid. In threonine aldolase from *Thermatoga maritima*, His-83 is thought to be the catalytic base catalyzing the proton abstraction from the β-hydroxyl of the substrate. Alanine racemace of *Geobacillus stearothermophilus* also contains an active site histidine (His-166). This residue forms a hydrogen bond with Tyr-265 and functions to lower its p*K*_a_ [31] and act as a base required to convert L- to D-alanine [32]. Removal of the hydrogen bond interaction between His-166 and Tyr-265 in the single Y265A variant of alanine racemase, resulted in an enzyme that exhibits aldolase activity with an apparent *k*_cat_/ *K*_m_ 2.3 × 10^5^-fold higher than racemase activity in the wild-type enzyme [33]. Racemase activity in the Y265A variant was reduced 4 × 10^5^-fold. The variant is also stereoselective, accepting only D-configured amino acids as substrates but displayed poor stereochemical control at the β carbon [34].

The enzyme 4-oxalocrontonate tautomerase (4-OT) catalyzes the conversion of 2-hydroxyhexa-2,4-dienedioate into 2-oxohex-3-enedioate in the degradation of aromatic hydrocarbons [35]. The enzyme belongs to the tautomerase superfamily that contain a unique catalytic amino-terminal proline. Recently it was discovered that the enzyme has a secondary activity in its ability to catalyze the aldol condensation between acetaldehyde and benzaldehyde with a *k*_cat_/ *K*_m_ of 8.5 × 10^-4^ M^-1^ s^-1^ to yield cinnamaldehyde [36]. In the tautomerase reaction, Pro-1 of 4-OT acts as a nucleophile to form an enamine intermediate. In the presence of electrophiles, this nucleophilic intermediate can undergo carbon-carbon bond formation reactions. Therefore the promiscuous aldolase activity of OT is presumed to result from the cross-coupling of acetaldehyde and benzaldehyde to yield 3-hydroxy-3-phenylpropanal which subsequently undergoes dehydration to yield cinnamaldehyde. It has been proposed that the final hydrolysis step is rate limiting. In an attempt to increase this step in the reaction, Poelarends and co-workers used previous knowledge of a F50A variant that increased the accessibility of the active site to the external aqueous environment without dramatic alterations to the p*K*_a_ of Pro-1 [37]. The resulting F50A variant exhibited a 600-fold increase in k_cat_/ *K*_m_ in the aldol condensation reaction of acetaldehyde and benzaldehyde.

Tyrosine phenol-lyase (TPL) is a PLP-dependent enzyme that is structurally similar to aspartate aminotransferase [38]. TPL catalyzes the β-elimination of L-tyrosine to produce phenol, pyruvate and ammonium. In an attempt to switch the substrate specificity of TPL from L-tyrosine to dicarboxylic amino acids, homology modeling indicated that Val-283 in TPL occupies the same position as Arg-292 in aspartate aminotransferase, a residue that binds the carboxylate group in the aspartate side chain. Arg-100 in TPL on the other hand corresponds to Thr-109 in aspartate aminotransferase, which interacts with the phosphate group of PLP. In the R100T/V283R double variant of TPL the β-elimination reaction towards dicarboxylic acids increase by at least 10^4^-fold compared to the wild-type enzyme and the rate of β-elimination using L-aspartate as substrate was 2-fold higher rate than that of L-tyrosine [39]. The rate of β-elimination was only one order of magnitude slower than that of L-tyrosine in the wild-type enzyme. Thus, the R100T/V283R variant was converted to a dicarboxylic amino acid β-lyase, an enzyme not found in nature without significant change to its reaction specificity.

**Figure 6:**
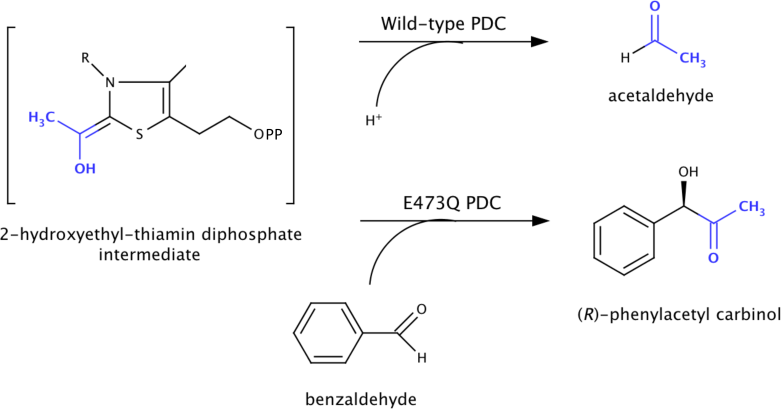
**Half reaction of pyruvate decarboxylase**. In the wild-type enzyme, protonation of the 2-hydroxyethyl-thiamin diphosphate intermediate leads to the release of acetaldehyde. In the E473Q variant, protonation occurs more slowly thus allowing for carboligation of benzaldehyde with the intermediate leading to the formation of (*R*)-phenylacetyl carbinol, a precursor of (-)-ephedrine.

The elimination of one catalytic function can also lead to the improvement of another secondary catalytic function. Pyruvate decarboxylase (PDC) is a thiamin diphosphate dependent enzyme that catalyzes the decarboxylation of pyruvate to form acetaldehyde and carbon dioxide in the presence of Mg^2 +^ (Figure 6) [40]. The enzyme is also able to catalyze a carboligation side reaction, whereby the carbanion/enamine intermediate, following decarboxylation of pyruvate, reacts with excess or exogenous aldehydes, to form 2-hydroxy ketones such as (*R*)-phenylacetyl carbinol, the precursor of several antiasthmatics such as ephedrine, pseudoephedrine, and norephedrine [41]. Kinetic and thermodynamic single-step analysis in conjunction with the X-ray crystal structure of PDC from *Zymomonas mobilis* implicated an active site residue Glu-473 in donating a proton to the 2-hydroxyethyl-ThDP carbanion/enamine intermediate to form acetaldehyde [42]. Consequently, in the E473Q variant, the protonation step occurs 2000-fold slower than the wild-type enzyme, and the catalytic cycle stalls at the carbanion/enamine intermediate state following pyruvate decarboxylation. Therefore this variant is more efficient at catalyzing formation of (*R*)-PAC in the presence of benzaldehyde [43]. The high enantioselectivity of the wild-type enzyme is retained in the variant.

## De novo rational computation design

The ultimate goal in rational protein design is the creation enzymes *de novo* i.e. creating synthetic protein catalysts. *De novo* design involves the selection of a catalytic mechanism for the desired reaction and modeling the reaction transition state(s). A protein scaffold is then designed to accommodate and stabilize the transition state. Usually several enzymes are produced, ranked and experimentally evaluated. C-C bond forming reactions are one of the best understood chemical reactions and not surprisingly some of the most successful examples of *de novo* designed enzymes are those that catalyze this reaction.

**Figure 7:**
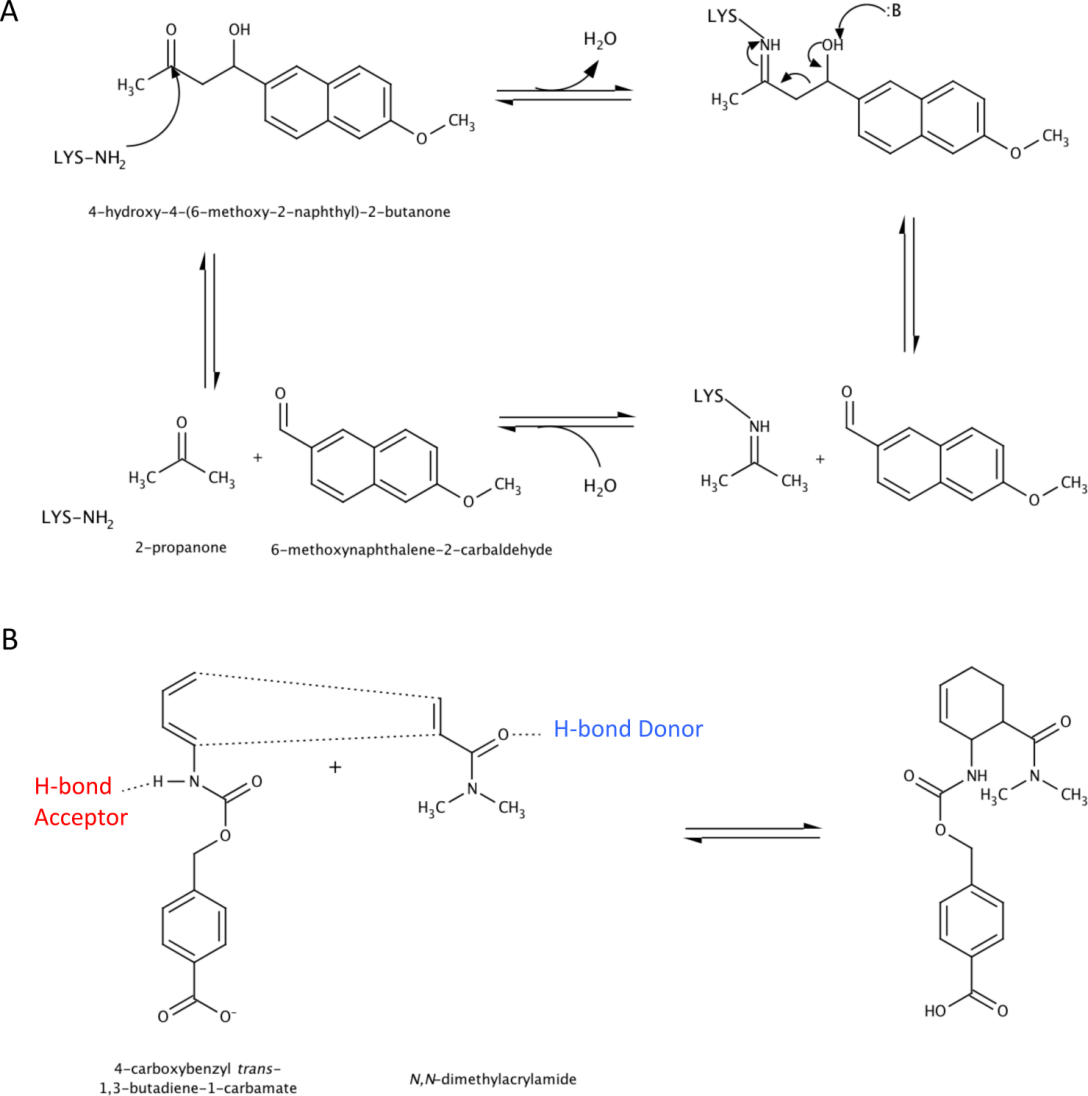
**Chemical reactions catalyzed by de novo designed enzymes**. (A) The retro-aldol reaction involves the formation of a Schiff base intermediate between a catalytic lysine and the substrate 4-hdyroxy-4-(6-methyoxy-2naphthyl)-2-butanone. C-C bond cleavage occurs following proton abstraction at the C4-OH of the substrate forming 2-propanone and 6-methoxynaphthalene-2-carbaldehyde. (B) The Diels-Alder reaction occurs between a dienophile I (4-carboxy *trans* 1,3-butadiene-1-carbamate) and diene II (*N*,*N*-dimethylacrylamide) which undergoes a pericyclic [4 + 2] cycloaddition to form a chiral cyclohexene ring.

Aldolases of the Class I type that form a Schiff base intermediate with a catalytic lysine [9, 44] were designed to catalyze the carbon-carbon bond cleavage of 4-hydroxy-4-(6-methoxy-2-naphthyl)-2-butanone, a compound not found in biological systems (Figure 7A) [45]. Four active site motifs with differing catalytic lysine environments, carbinolamine stabilization and proton abstraction were generated on five different protein scaffolds belonging to the triose phosphate-isomerase (TIM)-barrel and jelly-roll folds [46]. Of the 72 designs that were selected and experimentally characterized, 32 exhibited detectable aldolase activity. The most significant rate enhancement (*k*_cat_/ *k*_uncat_ of 2 × 10^4^) was accomplished through a catalytic lysine in a hydrophobic environment with a water molecule stabilizing the carbinolamine intermediate and catalyzing the proton abstraction of the C4-OH group of the substrate. Replacement of the putative catalytic lysine in all models dramatically decreased or abolished catalysis completely, suggesting that the observed activity was due to the formation of a schiff base intermediate with the substrate. Structures of two of the designed enzymes were solved, which showed that the active site residues superimpose well on the original design.

To date, no natural enzyme has been demonstrated to catalyze an intermolecular Diels-Alder reaction. Therefore *de-novo* rational design of an enzyme that catalyze a pericyclic [4 + 2] cycloaddition between a conjugated diene and a dienophile to form a substituted chiral cyclohexene compound, forming two carbon-carbon bonds and up to four new stereogenic centers in one step, was attempted. In the well-studied Diels-Alder reaction between 4-carboxybenzyl *trans*-1,3-butadiene-1 carbamate and *N*,*N*-dimethylacrylamide, the dominant interaction in the transition state is the highest occupied molecular orbital (HOMO) of the diene, 4-carboxybenzyl *trans*-1,3-butadiene-1 carbamate with the lowest unoccupied molecular orbital (LUMO) of the dienophile, *N*,*N*-dimethylacrylamide (Figure 7B). Thus, narrowing the energy gap between HOMO and LUMO is predicted to increase the rate of the reaction. An enzyme was devised whereby a hydrogen bond acceptor was positioned to interact with the carbamate NH of the diene, raising the energy of the HOMO energy while a hydrogen bond donor interacts with the carbonyl of the dienophile, lowering the LUMO energy. A binding pocket was also designed to position the substrates in the proper relative orientation by incorporating functional groups that can form hydrogen bonds with the substrates. Out of 207 stable protein scaffolds with backbone geometries that allow for orientation of the catalytic residues relative to the substrates, 84 designs were selected, 50 of which were soluble and can be purified [47]. Two enzymes with different scaffolds exhibited Diels-Alderase activity. The structure of one of these designs was solved using X-ray crystallography and showed agreement with the design model with a RMSD of 0.5 Å.

A challenge in *de novo* designed enzymes is that the protein backbone structure is not optimized for substrate binding or catalysis, resulting in low catalytic efficiencies. Directed evolution approaches are one way to circumvent the need for precise backbone modeling to enhance the catalytic efficiency of designed enzymes. An alternative approach is to enlist the aid of the internet community to perform time consuming human interpretation and judgment required for modeling of protein backbone structure. This has been successfully implemented in the crowdsourcing program foldit. Users (players) designed enzyme was found to improve the catalytic efficiency of the diels alderase mentioned above by up to 19-fold [48].

## Summary

The examples discussed above demonstrate that it is possible to alter the function carbon-carbon bond forming enzymes using knowledge based, rational approaches. Ultimately, such studies also strengthen our understanding of protein structure-function relationships that will in turn advance our ability to engineer proteins with desired functions with greater precision.
